# Some differences but all at risk: Improving farm safety for young people—An Australian experience

**DOI:** 10.3389/fpubh.2022.1031003

**Published:** 2022-11-08

**Authors:** Susan Brumby, Tracey Hatherell, Cecilia Fitzgerald, Jacqueline Cotton, Morna Semmens, Sally Cunningham, Jenna L. Gibbs, David Sullivan, Carolyn E. Sheridan

**Affiliations:** ^1^School of Medicine, Deakin University, Waurn Ponds, VIC, Australia; ^2^National Centre for Farmer Health, Western District Health Service, Hamilton, VIC, Australia; ^3^Ag Health and Safety Alliance, Greenville, IA, United States

**Keywords:** risk, schools, farm, gender, peers, safety, agriculture, young adults

## Abstract

A significant portion of on-farm deaths and injuries in Australia occur among young people working on the farm. Since most Australian farms are still family owned and operated, young people are an integral part of everyday operations and the farm is a place where these young people live, work and play. This paper describes how the international Gear Up for Ag Health and Safety™ program, originally developed in North America, was further developed for a younger Australian audience (ages 12–19) enrolled in agricultural programs at secondary or vocational schools. In addition, we share insight on demographics, self-reported farm safety behaviours, and the most common farm tasks being performed by program participants utilising a pre-survey originally developed for program customisation. Of particular importance were the most common farming tasks reported by this group. The most common tasks performed on Australian farms included a large variety of vehicle use (farm vehicles, motorbikes, and quadbikes) and handling livestock. Females reported operating vehicles and other farm equipment at the same rates as males. Males were more likely to be working with large heavy machinery and driving trucks, while females were more likely to be working with livestock and using horses for stockwork. Both males and females reported low use of PPE and poor safety habits. In future Australian programs, it will be important to address the conspicuous use of motor vehicles, quadbikes, motor bikes and machinery at early ages, and to target gender-specific tasks to reduce risks on the farm.

## Introduction

In Australia, agriculture, forestry and fishing has the highest rate of workplace fatality per 100,000 workers across all industries (1). The majority of these injuries are caused by vehicle crashes (quadbikes, motorbikes, tractors, utilities, trucks) and being hit by moving objects, including vehicles and machinery. According to a recent study by Peachey, from 2001 to 2019, 15% of all farm-related fatalities were youth under 15 years. Of these deaths, a third were visitors or bystanders at the farm and more than half had no active supervision by an adult (2). The major causes of both youth and adult deaths and injuries on farms are similar—quadbikes, tractors, farm vehicles, motorcycles; plus horses, contact with animals and water bodies for young people under 15 years (2, 3). Other occupational risks such as noise induced hearing loss and respiratory disease are also known to be higher in farming populations (4, 5) and linked with farm work exposures. Despite the known high-risk nature of agricultural vehicles, quadbikes and farming work, farmers and farming families continue to take risks on their farms.

The majority of Australian farms are family-owned and operated with farming families commonly described as families where at least one adult is a farmer or farm manager (6). Due to the family nature of farm operations, farms combine a functioning workplace and family home—that is they are where people live, work, and play. Due to this overlap of work life and home life, children and young adults are often exposed to a range of occupational hazards, and consequently learn much of their safety behaviours and attitudes from observing their parents, farm workers and siblings. Cigularov et al. (7) noted that young people (age 13–18 years) who are engaged in farm work and perceive a positive safety environment are more likely to communicate mistakes to their parents when the parents showed concern for safety. Peers (friends and school colleagues) can also be an important point of influence for shaping safety culture, although as noted by McBain-Rigg and colleagues this influence can have a positive or negative effect (8, 9). The role of the family and peers is important as previous research has shown a broad distrust of safety information coming from people with little or no farming experience making it difficult for safety professionals to provide meaningful input (10, 11).

Globally, most agricultural safety training programs and interventions are designed and targeted at younger people, aged 5–12 years (12, 13), and include a variety of formats such as the Progressive Agriculture Safety Day, Farm Safety 4 Just Kids or programs that are developed and delivered specifically in place such as the Hesse Farm Safety program (13, 14). However, whilst most programs show increases in knowledge, long term evaluations are limited and mostly remain unpublished (15, 16). The enduring Canadian primary school program “Safety Smarts”—running since 1998—showed that students increased their awareness and knowledge of farm hazards and built pro-safety attitudes that endured as they matured (17). A key success factor noted by the external evaluators was the first hand farm experience of all the facilitators which enabled trust building (17). Despite some success with primary school age programs, few studies globally have examined the impact of agricultural health and safety programs with young people aged 12–19 years old. As a result, there remains minimal evidence around what works. This is also reflected in Australia where there is a current gap in the provision and evaluation of engaging safety content for young adults (12–19 years) living and working in agriculture (2, 18).

A farm's safety culture is influenced by various factors including the culture of people on farms to act in a safe manner. However, these decisions, choices and attitudes around safety are shaped by family, schools, industry, community mores, and the policy of state and federal government legislative frameworks (8). For example in Victoria, Australia, under the Occupational Health and Safety Act 2004 (19), you need a permit to employ children under 15 years and must comply with the minimum age, hours of work and rest break requirements. Yet, for children working or helping on a farm (or other family business) these obligations do not apply as family businesses are exempt (20). This situation is common in many state and international jurisdictions, where family farms are treated differently to other businesses.

**Figure 1:**
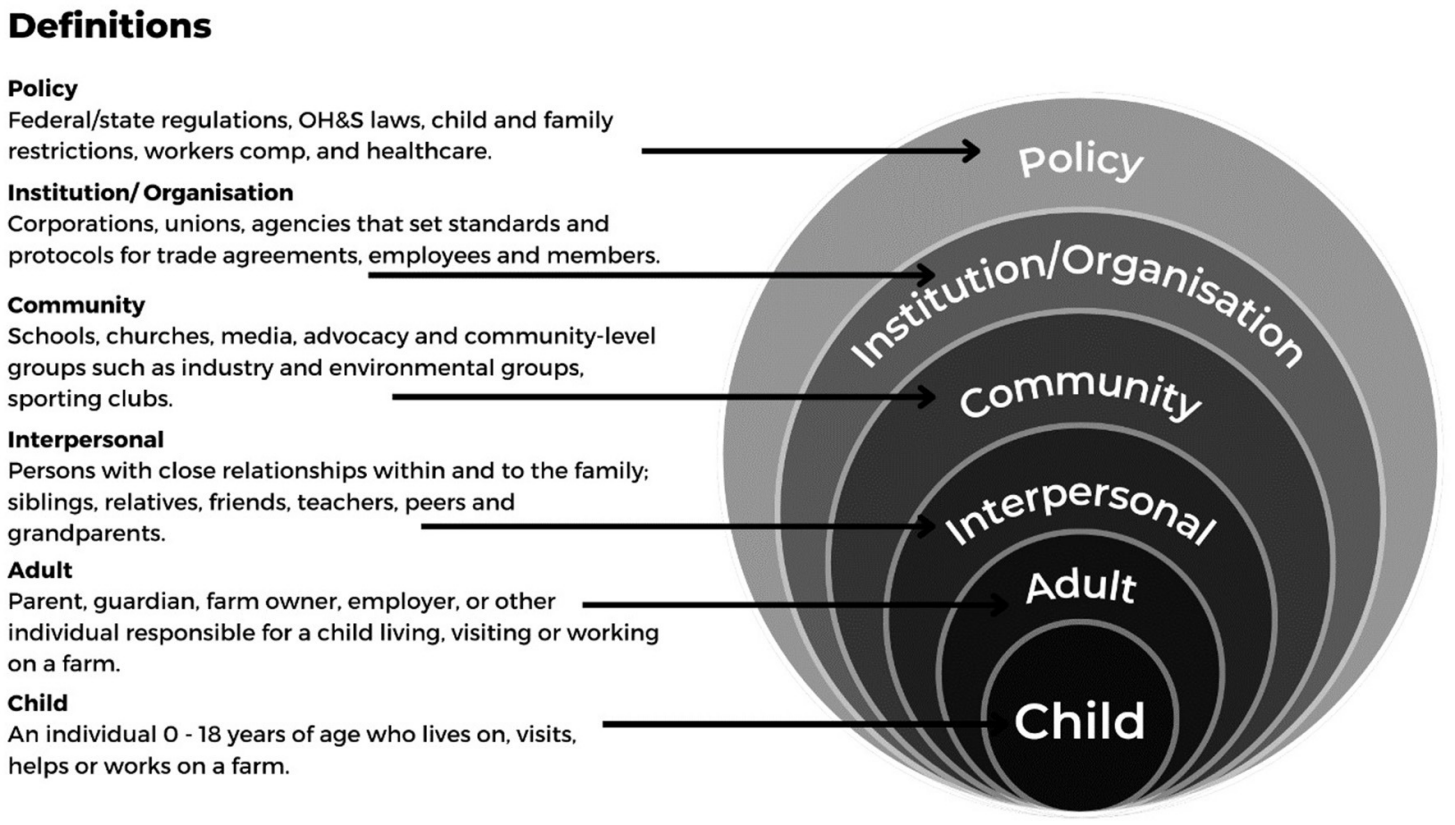
The socioecological model adjusted for young people and farm safety. Adapted from Lee et al. (23).

In understanding the broader elements that impact farm safety, Bronfenbrenner's socio-ecological model (SEM) (21) defined the five layers (self, interpersonal, community, organisational, and policy) which can each have an effect on a person's development. This interaction between self, family/community and school environment, and the broader political or government landscape shapes personal growth. Changes or conflict in any one layer can affect another (22). The SEM has been used previously to consider the broader issues in agricultural health and safety (8, 23, 24), with Lee et al. adding a sixth layer—child—as the first level (see Figure 1).

The international Gear Up for Ag Health and Safety™ program (Gear Up for Ag™) was designed to address a number of the key layers of the SEM framework (21). Developed by the Ag Health and Safety Alliance (AHSA), a non-profit organisation based in the US, Gear Up for Ag™ was developed to train 18–25 year olds in a range of agricultural health and safety topics (12).

This program was originally launched in the U.S. and Canada, and has trained more than 5,000 young adults in agriculture, globally. Preliminary findings have found that the program leads to shifts in knowledge on safety behaviours, especially those related to safe quadbike use, eye safety, hearing conservation, and sun protection. The training impacts participants, since most of them report being more aware of agricultural safety and health issues following the program and are very likely to engage in discussions about safety topics with their family members, coworkers, and peers. Up to one third of U.S. participants also state that they purchase additional safety equipment, such as PPE, following the program (12).

In 2018, experts from AHSA provided a train-the-trainer experience to assist in localisation and adoption of the Gear Up for Ag™ program pertinent to the Australian environment. The international visit and collaboration focussed on increasing local expertise for an interactive intervention to provide young people with evidence-based safety information about a range of agricultural health and safety topics. Following piloted programs in 2019, the Australian Gear Up for Ag™ program was further adapted to focus on a slightly younger audience, aged 12–19 years in secondary schools that were involved in agricultural studies or attended schools in farming communities.

## Materials and methods

The Gear Up for Ag™ education program is facilitated by Agricultural Health and Medicine graduates (25, 26) and/or industry representatives. All facilitators were required to have completed the train the trainer program and have a background in health (usually registered nurses), education and/or experience in farming or agricultural extension. A minimum of two facilitators were required to attend any Gear Up for Ag™ education program and ensure a combination of both health and agricultural expertise.

**Figure 2:**
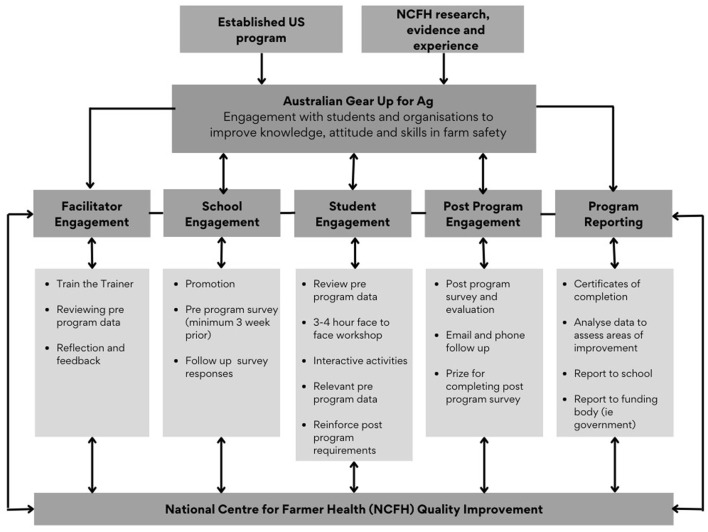
Method and process for delivering a Gear Up for Ag™ education program.

Figure 2 illustrates the five-stage process that is completed for each Gear Up for Ag™ education program. Student participants were from 13 secondary or vocational training organisations across the state of Victoria, Australia. The students may or may not have been enrolled in agricultural studies, but all attended school or vocational training [competency-based education (27)] in farming communities where they visit, work casually or help friends and family on farms. Gear Up for Ag™ was a useful introduction to the competency-based Certificate II/III courses. Several of the schools had their own small working farms with a variety of enterprises including horticulture to livestock where safety procedures were being taught and reinforced. Although the general purpose of the Gear Up for Ag™ program is to conduct educational and training outreach, the objective of this paper is to apply program evaluation techniques to share demographics, self-reported farm safety behaviours and analyse common farm tasks being performed including analysis by gender using SPSS 28 (28). It is hoped that this will help inform future delivery models and areas for wider analysis.

### Pre-program engagement

A key part of the program is engagement with the respective teachers. With cooperation and support of the school, a pre-survey of students attending the program is completed 2–3 weeks prior to commencement. This pre-survey ascertains the types of farming undertaken, age, and common activities and tasks students engage in on farm. Understanding of the common agricultural tasks and types of farming undertaken is very important to allow for refining of the content and tailoring to the audience. This contextualisation is crucial to the integrity and method of the program as farming industries/commodities are heterogeneous—the experience of a young person growing up and/or working on a dairy farm is very different to that on a cropping farm. A recent report into general safety interventions has urged education programs to consider the influence of context on interventions, noting that interventions are often “borrowed” from other organisations and not adjusted to meet specific needs (29). The pre-survey also identified current and contextual safety behaviours along with ascertaining if students were concerned for the safety of others such as families, or friends. In Australia, farmers are often presumed and promoted in social media to be male. As outlined by Brumby, it was important that all involved with the program were cognisant of not reinforcing or propagating stereotypes of male hegemony (30). To assist in shaping the material and understanding the role of gender and common farm tasks undertaken proportions were also calculated for common farm tasks undertaken in the last 12 months. A Two Sample *Z*-Test for Proportions was used to determine if tasks undertaken by males and females are different from one another and assess the relationship between farm tasks and gender.

### Face-to-face education program

A 3–4 hr education program is undertaken and includes general and specific information on agricultural hazards (as outlined in the pre-survey data), such as noise, machinery, livestock, large plant, motorbikes, quadbikes, tractors, horses, workshop equipment, dust, and agrichemicals. A mixture of giving information, undertaking discussion, reflection and interactive activities/ quizzes are utilised and practical advice on ways to work safely to minimise risk of injury and illness on farms. For example, an interactive activity using shaving cream to illustrate how agrichemical contamination on clothing and PPE can easily be transferred, ingested, and absorbed. Students often shared storeys of their experiences of farm accidents or injuries, identifying causes such as speed, no helmet, or seatbelt, and used language such as “unlucky,” and “done it before without a problem” (31).

### Post-program engagement

A post-survey is administered 4–6 weeks following the program. This time frame allows students to apply new knowledge, have safety discussions, and make possible changes in safety practises and behaviours to reduce risk. A particular focus is if the student had subsequent health and safety discussions with family, friends, or peers aligning with the influential factors described in the SEM framework of influences (21, 23). An incentive of going into a draw to win a half face respirator was offered to encourage survey completion.

A short 10 question survey is also sent to the responsible class teacher for completion post the Gear Up for Ag™ Program. This consists of six questions using a five point Likert (32) score based on communication, balance between activities and information and discussion, maintaining students interest, and applicability to the students work and life. Four open ended questions asked about any ongoing classroom discussion post-program and for recommendations for improvement.

The Deakin University Human Research Ethics Committee executive reviewed the Gear Up for Ag™ Health and Safety Evaluation (2021-256:) and found it to be compliant with the *Ethical Considerations in Quality Assurance and Evaluation Activities* guidelines (33) and no further ethics review was required.

## Results

From February 2021 to June 2022, there were 301 participant responses for the pre-survey. These were from students who attended the Gear Up for Ag™ education program. Demographics are presented in Table 1, along with whether they work on a farm, and supervise others.

**Table 1:** Demographics and safety habits of participating students pre-program (*N* = 301).

**Characteristics**	**Male** ***n* (%)**	**Female** ***n* (%)**	**Total** ***N* (%)**
Total study participants	162 (54)	149 (46)	301
Age (in years)
Mean (SD)	16.39 (3.80)	17.07 (5.60)	16.7 (4.84)
Age groups
13–15 years	66 (41)	60 (43)	126 (42)
16–19 years	84 (52)	66 (48)	150 (50)
≥20 years	12 (7)	13 (9)	25 (8)
What are you currently studying?			301
Agricultural science- secondary school	49 (30)	47 (34)	96 (32)
Certificate II or III in agriculture	65 (40)	58 (42)	124 (41)
Certificate II/III in animal studies	4 (2)	5 (4)	9 (4)
Other	44 (27)	29 (20)	72 (23)
Do you currently work and/or help on a farm?			301
Yes	125 (77)	91 (66)	216 (72)
No	37 (23)	48 (34)	85 (28)
What age did you start working on the farm?			301
4 years or younger	51 (31)	24 (17)	75 (25)
5–8 years	28 (17)	26 (18)	54 (18)
9–12 years	32 (20)	24 (17)	56 (19)
13–14 years	15 (9)	16 (12)	31 (10)
15 years or older	18 (11)	22 (16)	40 (13)
N/A I have never worked on a farm or in agriculture	18 (11)	27 (19)	45 (15)
Do you worry about the safety of family/friends on the farm?			301
Yes	111 (68.5)	106 (76)	217 (72)
No	44 (28)	26 (19)	70 (23)
I don't know anyone who works on a farm	7 (4)	7 (5)	14 (5)
How important do you think your own health and safety practises are on the farm?			301
Extremely important	81 (50)	76 (58)	157 (52)
Very important	49 (30)	40 (29)	89 (29)
Moderately important	24 (15)	18 (13)	42 (14)
Slightly important	3 (2)	2 (1)	5 (2)
Not at all important	5 (3)	3 (2)	8 (3)
Do you ride a quadbike on the farm?			301
Yes	113 (70)	84 (60)	197 (65)
No	49 (30)	55 (40)	104 (35)
Do you ever have passengers when riding on the quadbike?			197
Yes	70 (62)	48 (57)	118 (60)
No	43 (38)	36 (43)	79 (40)
Do you wear a helmet when riding a quadbike on the farm?			197
All the time	34 (30)	26 (31)	60 (30)
Most of the time	24 (21)	17 (20)	41 (21)
Occasionally	21 (19)	17 (20)	38 (19)
No I do not wear a helmet	34 (30)	24 (29)	58 (30)
Do you wear respiratory protection when working in dusty environments?			301
All the time	19 (13)	6 (4)	25 (8)
Most of the time	28 (27)	22 (16)	50 (17)
Occasionally	49 (30)	24 (17)	73 (24)
I do not wear respiratory protection	48 (30)	42 (30)	90 (30)
N/A I never work in dusty environments	18 (11)	45 (32)	63 (21)
Have you ever had short of breath, cough, fever or chills after being in a dusty environment?			238
Yes	36 (25)	31 (33)	67 (28)
No	107 (75)	63 (67)	171 (72)
In the past year have you been exposed to any loud noise on the farm?			301
Yes	128 (79)	91 (65)	219 (73)
No	34 (91)	48 (35)	82 (37)
In the past year have you mixed, handled or applied, agrichemicals on paddocks, livestock, in the dairy or around the home?			301
Yes	73 (45)	56 (40)	129 (43)
No	89 (55)	83 (60)	172 (57)
Of those currently working or helping on farm (*n* = 216)—Do you supervise others on the farm?	125 (57)	91 (43)	216
Yes	55 (34)	46 (33)	101 (47)
No	70 (43)	45 (32)	115 (53)
Who do you supervise? (may supervise either or both)			101
Siblings	45 (36)	37 (40)	82 (81)
Other workers	20 (16)	17 (19)	37 (37)

Most students were in their teens. Half the students were aged 16–19 years and 42% were 13–15 years old. The most common study being undertaken was the Certificate in Agricultural Studies (41%), followed by Agricultural Science as a secondary school subject (32%). Other courses included Victorian Certificate of Applied Learning (VCAL), and Certificate in Engineering with an agricultural focus.

Forty-five students (15%) had never worked on a farm with a third intending or wanting to. For those who had previously undertaken work or lived on a farm (*n* = 256), over half commenced at 8 years old or younger. Of those currently riding a quadbike on farm (*n* = 197), only half wear a helmet most or all the time and 60 per cent carry passengers. Reported quadbike behaviours were similar for both males and females. Seventy-two percent reported having been exposed to loud noise on the farm and 42 per cent had mixed, handled, or applied agrichemicals. Twenty-eight percent reported having experienced shortness of breath, fever, cough or chills after being in a dusty environment. Less than half wore respiratory protection most or all of the time in dusty environments. The majority (72%) indicated they worried about the safety of family or friends on the farm, and during the workshop some students articulated the implications of an injury or illness and subsequent effects of families (31). Over 80 per cent believed their own health and safety practises on the farm were important or extremely important. Of those currently helping or working on the farm (*n* = 216), 47% currently supervise others, with the majority being siblings and this was similar for males (36%) and females (40%).

**Figure 3:**
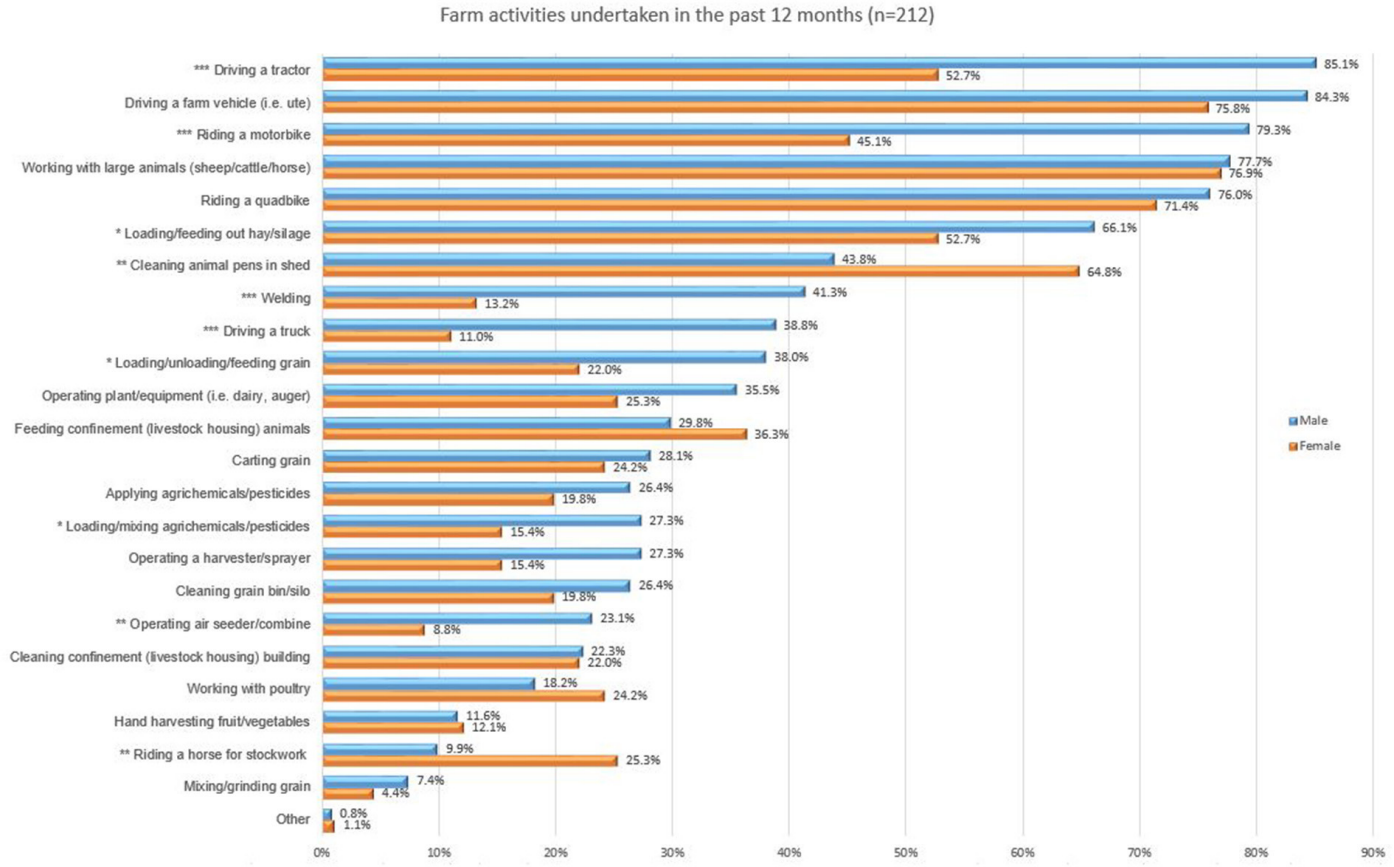
Proportion of participants reporting common tasks undertaken in last 12 months. *Significant difference between males and females using a Two Sample *Z*-Test for Proportions where ***p ≤ 0.001, **p ≤ 0.01, *p ≤ 0.05.

As shown in Figure 3, the most common farming tasks undertaken in the last 12 months included those most likely to injure or result in fatality, such as riding a quadbike, driving a tractor or other farm vehicle and working with large animals. Quadbikes in Australia have often accounted for the highest number of farm fatalities, having at times surpassed tractors (1). This is despite the guidelines from manufacturers and industry organisations that people under the age of 16 years should not ride quadbikes or take passengers (34).

A Two Sample *Z*-Test for Proportions (28) was used to determine if tasks undertaken by males and females are different from one another. The two-sample *Z*-test is a common method used to evaluate proportional differences between two groups of interest. Where there was a statistically significant difference this is shown with an asterisk on Figure 3.

Ten of thirteen schools responded to the stakeholder survey which was sent to the responsible subject teacher. Seven schools rated communication as excellent, six strongly agreed that the Gear Up for Ag™ program provided information and knowledge that added to their curriculum, and 100% agreed or somewhat agreed that the information was relevant to the students' lives, work or studies. All schools either strongly agreed or agreed that the program involved a good balance of information, hands-on activities, and discussion. Nine of ten schools who responded (90%) indicated that since the program, they had discussed some of the farm safety topics further with the students. Topics such as quadbike safety, biosecurity/zoonotic disease prevention, and agrichemical safety were the most likely to be discussed. All agreed they would recommend the Gear Up for Ag™ program to other schools.

There were 158 responses for the post-survey. A post-survey rate of 52% is a pleasing result, given the history of low response rates in other agricultural studies. Typically most survey response rates for academic studies of US farmers are ~20% (12, 35) and a recent study in Scandinavia found slightly higher results depending on whether email or post (36). On the post-survey, all students were given the opportunity to provide feedback in the form of a written, qualitative response. A third of the students reported that they or their families' had taken action to improve farm safety. Examples that were given included the fitting of roll over protection devices onto quadbikes, and the purchasing of additional personal protective equipment (PPE) such as safety goggles, respiratory masks, bike helmets, ear muffs, steel capped boots, chemical gloves and overalls.

## Discussion

Information on the tasks being performed by the students is important—not only to help inform and tailor future programs to the audience, but also to inform outreach messaging and future communication or safety campaigns. One of the major findings from this program is that vehicle use is a big component of farm work for the students and there is a heavy reliance on young people to undertake these tasks. Farm sizes in Australia are large with an average of 4,331 hectares, although farms in the state of Victoria are smaller (37). Due to the size and scale of farms the need for vehicle use (utilities, quadbikes, motorbikes) for transportation on the farm is required. This does make the farm environment in Australia different from other industrialised farming countries such as the US, UK, Canada and Europe where farm sizes are smaller (38).

Clearly the work undertaken on farm by young people is a core and key activity to the farm business. As shown in Figure 3, the most common tasks reported included those most likely to injure or kill on farm, such as driving farm vehicles, riding motorbikes and quadbikes (1, 2). This is despite the guidelines from manufacturers indicating that people under the age of 16 years should not ride quadbikes. The wearing of helmets was particularly low in those riding quadbikes, with only half wearing helmets most or all the time. Interestingly a higher rate of helmet wearing was noted for those riding motorbikes with 78% wearing helmets most or all the time. Additionally, the carrying of passengers on quadbikes is not recommended at any time for adults, let alone for young people under 16 years who should not be using the quadbike anyway (34).

Traditionally farm work, media and safety campaigns have seen a strong male bias, simply because most farmers were assumed to be males, distorting the reality of farming for both men and women (39, 40). As the results demonstrate, this is not the case, with females riding quadbikes, driving farm utilities, operating plant and equipment and working with large animals at the same rates as males. However, there were differences noted between genders as shown in Figure 3. Whilst males were significantly more likely to be working with large heavy machinery and driving trucks, females were significantly more likely to be working with livestock (including handling large animals) and using horses for stockwork. These task-based results were similar to those reported among young adults enrolled in the U.S. Gear Up for Ag™ program (12). Horses are the major cause of injury and death from animals in Australia (2, 3), and one area where there is significant differences between the genders. A recent paper by Shisler and Sbicca (41), highlights the variety of predominantly feminine-coded work on farms—such as feeding, stockwork and working with animals and explores the space of carework and farming and an expanded role beyond the masculine ideal. Females are doing significantly more “cleaning of pens” than males, and highlight the opportunity for future education programs to focus on proper PPE for cleaning campaigns and feature females performing the work, including driving vehicles, stockwork on horses, operating plant and equipment and cleaning pens.

The reported rates of hearing protection use (always or mostly) in noisy situations (32%) and use of respiratory protection (always or mostly) in dusty conditions (25%) by the students was low. As a comparison, 81% of young adults enrolled in the U.S. programs reported wearing hearing protection always or mostly (12). This difference between nations is concerning, especially since noise induced hearing loss is high in farming populations in Australia and the low rate of hearing protection use is concerning as once damage is done, it is not repairable (42–44). These findings highlight a significant lack of safety behaviours and the need to undertake further work with students to protect against occupational risks.

Finally, this work has highlighted that 47% of secondary school students supervise their siblings in the most hazardous workplace in Australia. This major finding highlights an opportunity to consider the key role of siblings, family and the broader community and industry in which agriculture and farming sit. As shown, there remains room for improvement in both awareness and practise of basic farm safety habits and those who are supervising younger siblings can assist in improving the culture for all. Importantly 52 students had discussed safety with their family post-program and also taken action to improve safety.

Future steps to address the findings of this program highlight the role of using schools to educate young people on farm safety as a valuable yet an underutilised component to the broader influence of safety culture. As highlighted by the elements from the SEM (21), it is also important that education provided external to the farm environment such as schools, peer based educators, and community based materials is consistent with safety messaging received on-farm from the primary farm worker and industry (45). For example the recent Victorian Farmers Federation's (VFF) “Making Our Farms Safer” campaign provides safety education and resources for farm owners, parents and peer groups (46). There is also a need for policy and strategic partnerships to support the expansion and availability of programs and educational content targeting children of primary school age (under 11 years) in order to see safety messaging “reinforced” rather than “introduced” once youth reach their teen years. Additionally, the impact of any educational programs and resources requires consistent and sustained evaluation to determine the effect youth education programs are having on the indicators of farm safety culture.

## Limitations

There is always the potential of bias when relying on self-reported surveys. One way that we addressed the bias is that the pre-survey occurred in a time frame that was before the Gear Up for Ag™ program was delivered onsite, so there was less potential to influence student responses. In addition, the post-survey and stakeholder survey was administered 4–6 weeks post-program, allowing people time to think about it. One limitation to the survey method is that although the same participant group completed the pre- and post-surveys, these responses are not paired. Numbers in the study were smaller (for example, 301 responses to the pre-survey) and prohibited more complex analysis of demographic factors and survey responses. However, the Gear Up for Ag™ program is focused around improving educational programs through outreach and were not originally designed to conduct research and instead are primarily used for program customisation is not necessarily a research program.

## Conclusion

The Gear Up for Ag™ program has highlighted not only areas of risk for individual students but the broader safety risks to siblings, peers, parents, schools and the farming community. Addressing the very common use of vehicles, machinery and equipment at an early age and the targeting of gender specific tasks to reduce risk must be considered in the development and delivery of any future programs and policy shifts. The Gear Up for Ag™ program has also illustrated the intrinsic value of discussion and the importance of subsequent student to family conversation to prompt safety action. Giving young people the language and tools to think about safety and articulate concerns is empowering for any young person whether off or on the farm.

## Data availability statement

The raw data supporting the conclusions of this article will be made available by the authors, without undue reservation.

## Ethics statement

The studies involving human participants were reviewed and approved by the Deakin University Human Research Ethics Committee. Written informed consent for participation was not provided by the participants' legal guardians/next of kin because: Deakin University Human Research Ethics Committee executive reviewed the Gear Up for Ag™ Health and Safety Evaluation (2021-256:) and found it to be compliant with the Ethical Considerations in Quality Assurance and Evaluation Activities guidelines.

## Author contributions

SB, CS, and DS contributed to the original piloting of Gear Up for Ag in Australia. TH and CF conducted the data collection. MS and SC the delivery of programs. TH and SB undertook the data analysis. SB, JC, and JG the drafting of the manuscript. JC, TH, and CF provided guidance and supervision throughout the study. All authors contributed to the study design, reviewing and editing of the manuscript, and approved the final manuscript.

## Funding

The Gear Up for Ag™ Program in Australia was piloted by the National Centre for Farmer Health, Victoria, Australia in 2019 and in 2020 received funding from Smarter, Safer Farms, Department of Agriculture, Victoria. Some donations of PPE were received from Big Safety for use in the pilot program.

## Conflict of interest

Authors JG, DS, and CS were employed by Ag Health and Safety Alliance. The remaining authors declare that the research was conducted in the absence of any commercial or financial relationships that could be construed as a potential conflict of interest.

## Publisher's note

All claims expressed in this article are solely those of the authors and do not necessarily represent those of their affiliated organizations, or those of the publisher, the editors and the reviewers. Any product that may be evaluated in this article, or claim that may be made by its manufacturer, is not guaranteed or endorsed by the publisher.
